# Luteolin Reduces Aqueous Extract PM2.5-induced Metastatic Activity in H460 Lung Cancer Cells

**DOI:** 10.7150/ijms.73947

**Published:** 2022-08-21

**Authors:** Hui-Wen Lin, Ting-Jing Shen, Nae-Cherng Yang, Meilin Wang, Wen-Che Hsieh, Chen-Ju Chuang, Chane-Yu Lai, Yuan-Yen Chang

**Affiliations:** 1 Department of Optometry, Asia University, Taichung, Taiwan.; 2 Department of Medical Research, China Medical University Hospital, China Medical University, Taichung 40402, Taiwan.; 3 Department of Microbiology and Immunology, School of Medicine, Chung-Shan Medical University, and Clinical Laboratory, Chung Shan Medical University Hospital, Taichung, Taiwan.; 4 Department of Nutrition, Chung Shan Medical University and Chung Shan Medical University Hospital, Taichung, Taiwan.; 5 Chinese Medicine Department, Ditmanson Medical Foundation, Chia-Yi Christian Hospital, Chia-Yi, Taiwan.; 6 Emergency department, Kaohsiung Municipal United Hospital, Kaohsiung, Taiwan.; 7 Department of Occupational Safety and Health, Chung Shan Medical University, Taiwan; Department of Occupational Medicine, Chung Shan Medical University Hospital, Taichung, Taiwan.

**Keywords:** luteolin, PM2.5, lung cancer, metastasis, EGFR-PI3K-AKT signalling

## Abstract

Fine particulate matter (PM2.5) is the critical cause of lung cancer and can further promote tumor cell migration and invasion. This study investigated the effects of luteolin, an antiangiogenic flavonoid agent, on blocking aqueous extract PM2.5-prompted cancer progression. We observed that luteolin reduced cell migration and the expression of pro-metastatic factors pro-matrix metalloproteinase (MMP)-2 and intercellular adhesion molecule (ICAM)-1 in PM2.5-exposed H460 lung cancer cells. Luteolin treatment also reduced the transduction of PM2.5-induced epidermal growth factor receptor (EGFR)-phosphatidylinositol 3-kinase (PI3K)-protein kinase B (AKT) cascade signaling. Furthermore, the reduction of MMP-2 expression and ICAM-1 production by luteolin in PM2.5-stimulated H460 cells is EGFR-PI3K-AKT pathway dependent. These results suggest that luteolin exhibits antitumor progression by inhibiting EGFR-PI3K-AKT pathway.

## Introduction

Ambient particulate matter (PM) and outdoor air pollution are classified as Class I human carcinogens by the International Agency for Research on Cancer 1. Fine particulate matter (PM2.5), a toxic air pollutant, has considerably increased the incidence of asthma, chronic obstructive pulmonary disease, cardiovascular diseases, and cancer progression by inducing intracellular oxidative stress, mutagenicity/geneotoxicity, and inflammatory responses 2. According to the comparative risk assessment of Global Burden of Disease 2010, PM2.5 causes ~3.2 million premature deaths worldwide in 2010 3. Moreover, Cohen et al. report that there are ~4.2 million deaths attributable to ambient PM2.5 in 2015 4, indicating that the threat of PM2.5 is increasing in human life globally. Therefore, it is urgent to find a possible treatment to reduce the PM2.5-attributed diseases.

Studies have shown that PM2.5-stimulated human non-small cell lung cancer (NSCLC) cells exhibit marked epithelial-mesenchymal transition (EMT) morphological changes. The expressions of EMT markers such as matrix metalloproteinase (MMP)-2 and MMP-9, which degrade the extracellular matrix, have also been reported to be elevated in PM2.5-exposed cells 5-7. In addition, PM2.5 facilitates the expression of intercellular adhesion molecule (ICAM)-1, a metastatic factor, in pulmonary epithelial A549 cells and rat plasma 8, 9. These studies suggest that PM2.5 exacerbates cancer cell migration and invasion, although the detailed mechanisms underlying this effect are not fully understood. However, considerable efforts are being made to develop strategies to prevent tumor cell invasion under PM2.5 exposure.

Natural products such as flavonoids are widely used to modulate several biological signaling pathways that are involved in the management of cancer. Many compounds derived from flavonoids are further applied in clinical studies of cancer therapy, such as catechins, quercetin, and luteolin 10. Luteolin (3',4',5,7-tetrahydroxyflavone), which is abundant in artichokes, celery, chamomile, green peppers and many medicinal herbs 11, has remarkable biological effects, including antioxidant, anti-angiogenic, anti-microbial, anti-allergic, and anti-inflammatory effects 12. A study showed that luteolin hinders tumor progression by suppressing kinases, regulating the cell cycle, inducing cell apoptosis and death, and reducing transcription factors 13. Notably, luteolin is reported to reduce not only vascular inflammation by inhibiting tumor necrosis factor (TNF)-α-induced ICAM-1 production, but also interleukin (IL)-1β-stimulated MMP-9 and MMP-13 expression 14, 15. Collectively, these reports have suggested that luteolin inhibits tumor progression.

In this study, we found that luteolin inhibited not only the ICAM-1 and MMP-2 production, but also the cell migration ability. Luteolin treatment also reduced the PM2.5-activated epidermal growth factor receptor (EGFR)-phosphatidylinositol 3-kinase (PI3K)-protein kinase B (AKT) signaling transduction in human H460 lung cancer cells.

## Materials and Methods

### PM2.5 samples preparation

PM samples were collected between August 2017 and May 2018 in the South District of Taichung City using the high-volume cascade impactor (TE-231, Tisch Environmental, Cleves, Ohio. USA) and the high-flow sampler (TE-6070, Tisch Environmental, Cleves, Ohio. USA). Aerodynamic diameter < 2.5 μm (PM2.5) of the particulates were harvested using 8” × 10” quartz filters (Pall, USA) through an inertial particle separator. Then, according to previous studies, the PM samples were analyzed 16, 17. Seventeen polycyclic aromatic hydrocarbons (naphthalene, acenaphthylene, acenaphthene, fluorene, phenan-threne, anthracene, fluoranthene, pyrene, benz(a)anthracene, chrysene, ben-zo(b)fluoranthene, benzo(k)fluoranthene, benzo(e)pyrene, benzo(a)pyrene, indeno (1,2,3-cd)pyrene, dibenz(ah)anthracene, and benzo(ghi)perylene) were quantified by gas chromatography with a flame ionization detector (GC-FID) using a Perkin Elmer Autosystem Gas Chromatograph (Agilent 7890B GC/5977 MSD) with a capillary column (50 m × 0.32 mm × 0.17 μm, Hewlett Packard). Twenty-two metals (Ga, Ag, Cd, Sn, Ba, B, N, Ni, Co, Cr, As, Se, In, Hg, Pb, Cu, Al, Ca, Zn, Fe, Mg, and Mn) were quantified using a flame atomic absorption spectrometer (PerkinElmer/NexION 300X, USA). The components analysis of PM2.5 samples were shown in the Supplementary table 1. The PM 2.5 solution was prepared in ultrapure water and stored at -20 °C. Before use, PM2.5 samples were sonicated for 3 minutes to ensure the homogeneity of the solution.

### Cell culture

NSCLC H460 cells were cultured in Dulbecco's Modified Eagle's medium containing 5% heat-inactivated fetal bovine serum (Cat#10437, Gibco), 1 mM sodium pyruvate, 1 mM nonessential amino acid, and 100 ng/mL penicillin/streptomycin (Cat#15140, Gibco) at 37 °C in 5% CO_2_.

### Agents and antibodies

Luteolin (Cat#L9283) was purchased from Sigma Co. (St. Louis, MO, USA). LY294002 was purchased from Cayman Chemical (Ann Arbor, MI, USA). Antibodies against MMP-2, EGFR, and PI3K-p85 were purchased from Spring Bioscience. Antibody against p-AKT^Ser473^ was purchased from Santa Cruz Biotechnology Inc. (Santa Cruz, CA, USA). The antibody against GAPDH was purchased from Taiclone. The lactate dehydrogenase (LDH) detection kit was purchased from Promega (Promega, Madison, WI, USA). The Cell Counting Kit-8 (CCK-8) was purchased from Dojindo Molecular Technologies, Inc. (Rockville, MD, USA).

### Zymography assay

Cells were collected with sample buffer (without 2-mercaptoethanol and dithiothreitol) and loaded on 8% SDS polyacrylamide gel containing 0.1% gelatin. The samples in the gel were electrophoresed at 120 volts for 120 minutes. Then, the gel was washed 3 times with wash buffer (2.5% Triton X-100 in distilled water) and distilled water. Next, the gel was incubated with 100 mL of reaction buffer at 37 °C for 24 h. After the reaction buffer was removed, the gel was stained with Coomassie Brilliant Blue R-250 at room temperature. Thirty minutes later, distilled water was added to destain the gel until the bands on the gel became clear. Then, the expressions of the indicated factors on the gel were imaged and analysed.

### Enzyme-linked immunosorbent assay (ELISA)

Supernatants from the cell culture were collected at the indicated time point. The concentration of human ICAM-1 was determined using an ELISA kit. All procedures were conducted according to the manufacturer's instructions (Cat#900-K464, PeproTech).

### Wound healing assay

Cells (8 × 10^4^ cells/100 μl/well of the insert) were seeded in the 2-well culture insert in 12-well plate. Next day, the culture-insert was removed and cells were pretreated with luteolin (30 μM) for 1 h following stimulated by PM2.5 (100 μg/ml) for 24 h. The images of cell migration were photographed by an inverted microscope.

### Western blotting

Cells were harvested and extracted with lysis buffer containing protease inhibitors (Sigma-Aldrich). After incubation on ice for 15 minutes, the samples were mixed with 4 × protein loading dye and heated to 100 °C for 15 minutes. Next, proteins were separated through sodium dodecyl sulfate polyacrylamide (SDS) gel electrophoresis and transferred to a polyvinylidene difluoride (PVDF) membrane (Millipore). The membrane was blocked with 5% nonfat milk in phosphate-buffered saline-buffered saline containing 0.05% Tween-20 (PBS-T) at room temperature for 1 h and then washed 3 times with PBS-T. Tar-get proteins were immunolabeled with indicated primary antibodies overnight at 4 °C. Horseradish peroxidase (HRP)-conjugated secondary antibodies were then used to detect the primary antibodies-protein complexes at room temperature for 1 h. Then, the protein-antibody complexes with HRP on the PVDF membrane were detected using an enhanced chemiluminescence western blot detection kit. The signals were captured with an imaging system.

### Statistical analysis

One-way ANOVA (Tukey's multiple comparisons test) was used for comparisons among more than 3 groups. Values are expressed as means ± standard error of the mean (SEM). All *P* values were calculated from two-tailed tests of significance. A *P* value of <0.05 was considered statistically significant.

## Results

### Luteolin Reduces PM2.5-induced Metastasis

**Figure 1:**
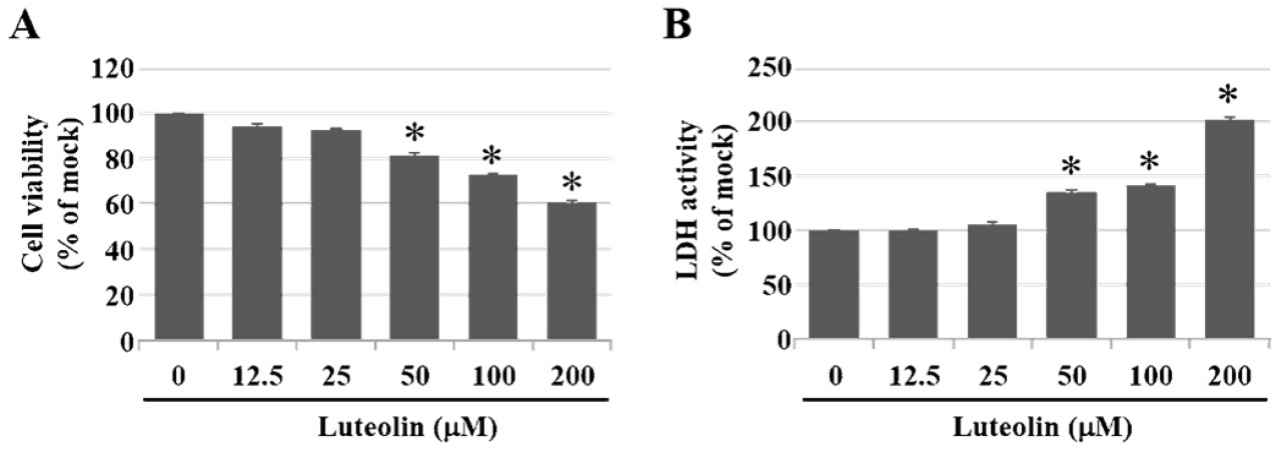
** Luteolin increases cell mortality and cytotoxicity in a dose-dependent manner of H460 cells.** H460 cells were treated with luteolin at doses of 0 (mock), 12.5, 25, 50, 100, or 200 µM for 24 h. **(A)** Cell viability and **(B)** cytotoxic effects of different doses of luteolin on H460 cells were analysed using a CCK-8 and LDH assay, respectively. Cells without luteolin treatment were used as the control group (mock). Quantitative data are presented as the mean ± SEM of the experiments (n = 3). ^*^P < 0.05, significant compared to the mock group.

**Figure 2:**
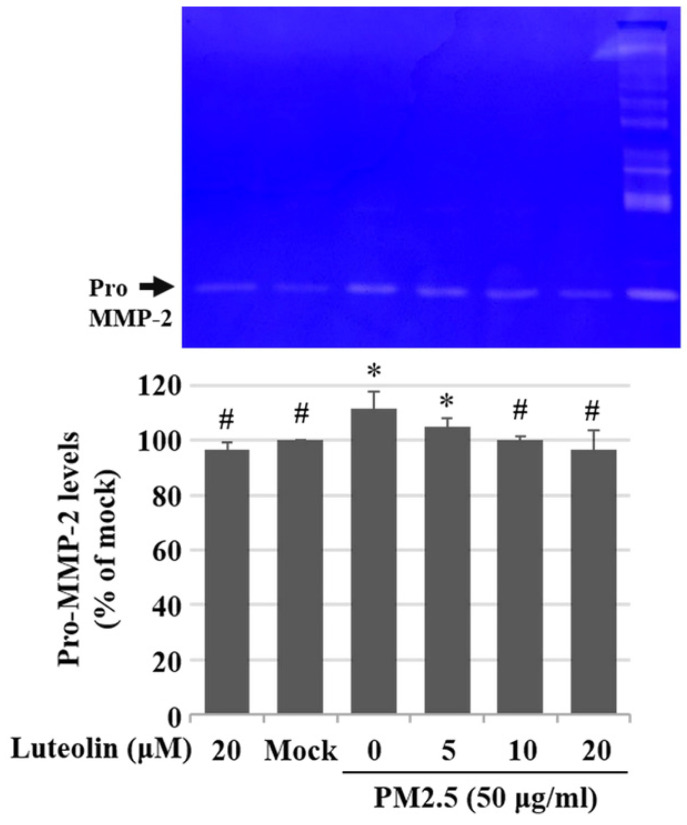
** Luteolin reduces aqueous extract PM2.5-enhanced pro-MMP-2 activity.** H460 cells were pretreated with luteolin (0, 5, 10, or 20 µM) for 1 h and then stimulated with PM2.5 (50 µg/ml) for 48 h. Zymography assay showed pro-MMP-2 levels. Cells without PM2.5 stimulation and luteolin treatment were used as the control group (mock). Quantitative data are presented as the mean ± SEM of the experiments (n = 3). ^*^P < 0.05, significant compared to the mock group; ^#^*P* < 0.05, significant compared to the PM2.5-exposed cells without luteolin treatment.

**Figure 3:**
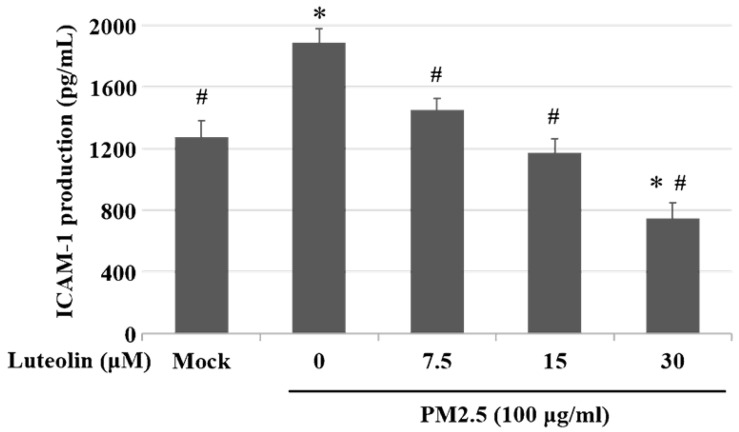
** Luteolin inhibits the aqueous extract PM2.5-induced ICAM-1 production.** H460 cells were pretreated with luteolin (0, 7.5, 15, or 30 µM) for 1 h and then stimulated with PM2.5 (100 µg/ml) for 24 h. ELISA was used to detect ICAM-1 production. Cells without PM2.5 stimulation and luteolin treatment were used as the control group (mock). Quantitative data are presented as the mean ± SEM of the experiments (n = 3). ^*^P < 0.05, significant compared to the mock group; ^#^*P* < 0.05, significant compared to the PM2.5-exposed cells without luteolin treatment.

**Figure 4:**
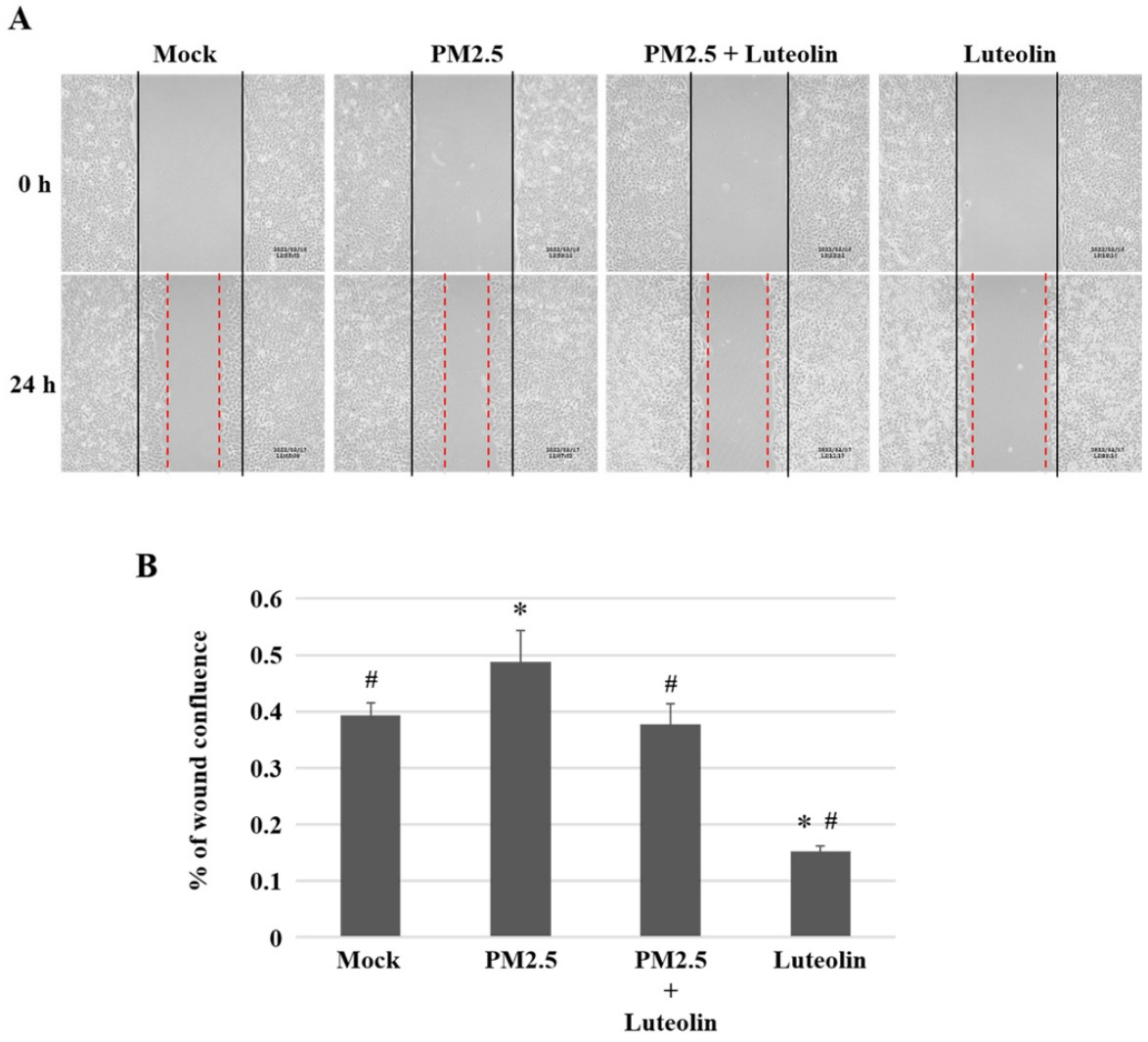
** Luteolin decreases the ability of cell migration in H460 cells.** H460 cells in 2-well culture-insert were pretreated with luteolin (30 µM) for 1 h then stimulated with PM2.5 (100 µg/ml) for 24 h. **(A)** The images of cell migration were photographed by an inverted microscope. Dashed lines measured the distance of the wound. **(B)** The statistical results of (A). Cells without PM2.5 stimulation and luteolin treatment were used as the mock group. Quantitative data are presented as the mean ± SEM of the experiments (n = 3). ^*^P < 0.05, significant compared to the mock group; ^#^*P* < 0.05, significant compared to the PM2.5-exposed cells without luteolin treatment.

Inhalation of PM2.5 increases the incidence of pulmonary diseases; specifically, it exacerbates lung cancer metastatic activity 18. To inhibit lung cancer progression, we treated human H460 lung cancer cells with luteolin (0 (mock), 12.5, 25, 50, 100, or 200 μM), a potent anticancer agent 11. Cell mortality (Figure 1A) and cytotoxicity (Figure 1B) were increased in H460 cells treated with luteolin at a dose greater than 50 μM. After administering a safe dose of luteolin, we observed that the activity of metastatic factor pro-MMP-2 was increased by aqueous extract PM2.5 stimulation but reduced by luteolin treatment (0, 5, 10, or 20 μM) in H460 cells (Figure 2). Luteolin treatment also limited the production of PM2.5-stimulated prometastatic ICAM-1 (Figure 3). Furthermore, the images (Figure 4A) and statistical results (Figure 4B) from *in vitro* wound healing assay showed that luteolin reduced PM2.5-prompted cell migration. These findings indicate that luteolin promotes metastatic progression in H460 lung cancer cells.

### Luteolin Decreases EGFR, p-PI3K, and p-AKT Expressions

**Figure 5:**
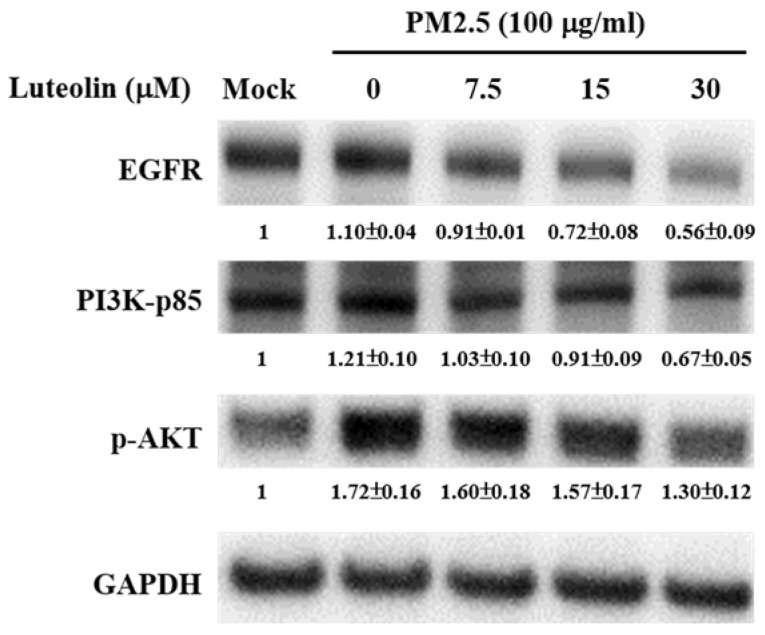
** Luteolin downregulates the protein expressions of the EGFR-PI3K-AKT cascade in aqueous extract PM2.5-exposed H460 cells.** H460 cells were pretreated with luteolin (0, 7.5, 15, or 30 µM) for 1 h and then stimulated with PM2.5 for 24 h. Western blot showed the protein expressions of EGFR, PI3K-p85, p-AKT, and GAPDH. Cells without PM2.5 stimulation and luteolin treatment were used as the control group (mock). Quantitative data are presented as the mean ± SEM of the experiments (n ≥ 3).

EGFR is highly expressed in patients with NSCLCs, and the downstream PI3K-AKT signalling activation drives lung cancer angiogenesis, invasion, survival, and metastasis 19-22. H460 cells were pretreated with luteolin (0, 7.5, 15, or 30 μM) for 1 h after stimulation with aqueous extract PM2.5 at a dose of 100 μg/mL for 24 h. Western blot analysis revealed that the protein expressions of PM2.5-increased EGFR, PI3K-p85, and p-AKT were reduced by luteolin treatment in a dose-dependent manner (Figure 5). These results indicate that luteolin participates in downregulating PM2.5-activated EGFR-PI3K-AKT pathway.

### The Inhibition of Luteolin on PM2.5-prompted MMP2 and ICAM-1 Expressions Requires EGFR-PI3K-AKT Signaling

**Figure 6:**
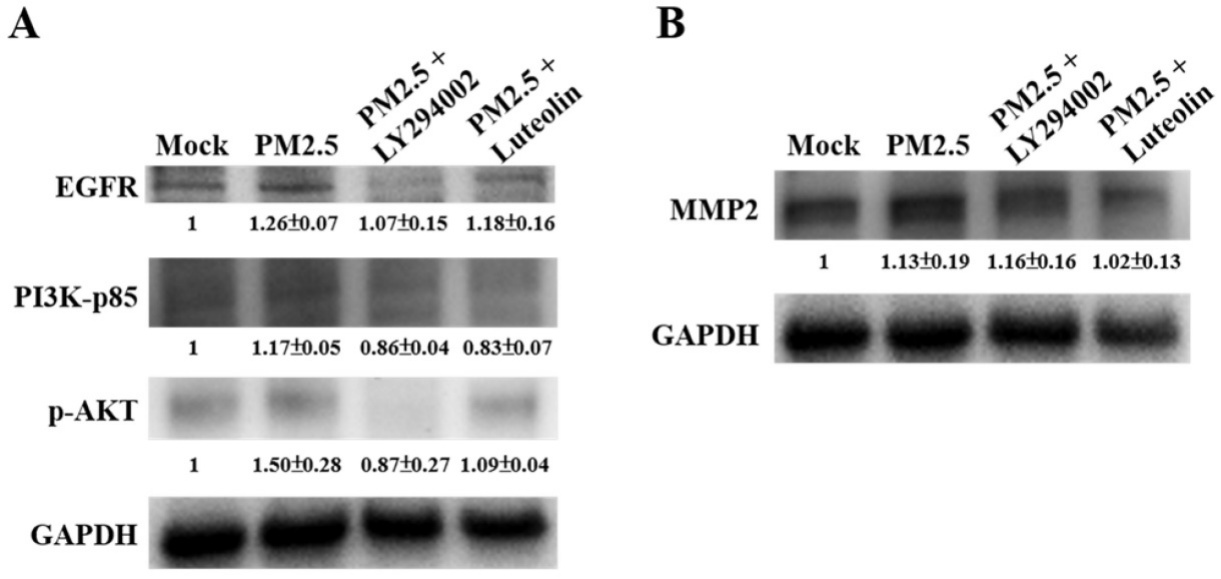
** The EGFR-PI3K-AKT pathway is critical for luteolin blocking the aqueous extract PM2.5-induced MMP2 activation.** H460 cells were pretreated with LY294002 (PI3K/AKT inhibitor, 20 µM) or luteolin (30 µM) for 1 h following stimulated with PM2.5 (100 µg/ml) for 24 h. Western blot showed the protein expressions of **(A)** EGFR, PI3K-p85, p-AKT, **(B)** MMP2, and GAPDH. Cells without PM2.5 stimulation and blockers were used as the control group (mock). Quantitative data are presented as the mean ± SEM of the experiments (n ≥ 3).

**Figure 7:**
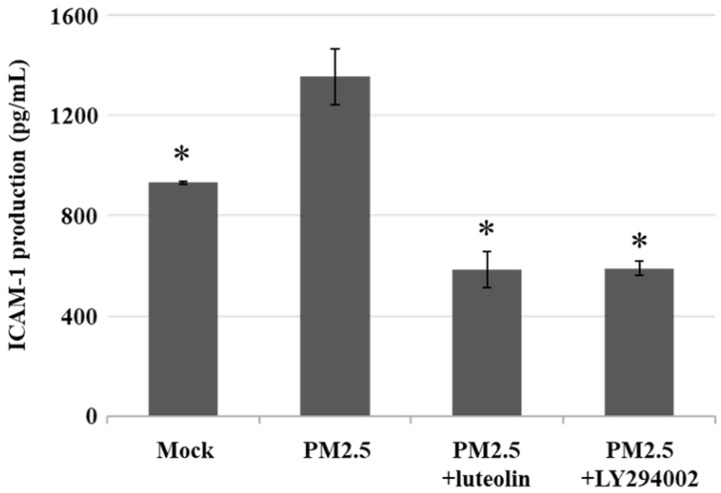
** Luteolin and PI3K/AKT inhibitor reduce the production of aqueous extract PM2.5-induced ICAM-1.** H460 cells were pretreated with luteolin (30 µM) or LY294002 (20 µM) for 1 h following stimulated with PM2.5 (100 µg/ml) for 24 h. ELISA was performed to detect the ICAM-1 production. Cells without PM2.5 stimulation and agent administration were used as the control group (mock). Quantitative data are presented as the mean ± SEM of the experiments (n = 3). *P < 0.05, significant compared to the mock group.

Next, to compare the effects of luteolin on PI3K-AKT signaling transduction, we pretreated H460 cells with the PI3K/AKT inhibitor LY294002 (20 μM) or luteolin (30 μM) for 1 h then stimulated the cells with aqueous extract PM2.5 at a dose of 100 μg/mL for 24 h. Western blot revealed that protein expressions of EGFR, PI3K-p85, and p-AKT were increased by PM2.5 exposure but reduced by LY294002 and luteolin treatment of the H460 cells (Figure 6A), showing that luteolin can inhibits PI3K-AKT pathway under PM2.5 stimulation. Based on previous results of this study that luteolin could reduce aqueous extract PM2.5-induced pro-MMP-2 activity (Figure 2) and ICAM-1 production (Figure 3), we next investigated the effects of luteolin on PI3K-AKT signaling transduction in PM2.5-induced MMP-2 protein expression. After 1 h of pretreatment with LY294002 (20 μM) or luteolin (30 μM), H460 cells were stimulated with 100 μg/mL of PM2.5 for 24 h. Western blot represented that the protein expression of MMP-2 was reduced by luteolin and LY294002 treatments in PM2.5-stimulated H460 cells (Figure 6B). Moreover, the administration of luteolin revealed comparable capacity with the treatment of LY294002 in reducing PM2.5-induced ICAM-1 production (Figure 7). These results suggest that EGFR-PI3K-AKT pathway is crucial for luteolin to inhibit PM2.5-promoted cancer metastasis.

## Discussion

Environmental carcinogenic factors, such as UV, heavy metals, and food additives, can lead to strong oxidative stress that results in various cancers 23. The fine PM2.5 exacerbates inflammation and diseases such as diabetes mellitus, cardiovascular diseases, and respiratory system injuries through various pathways and factors. Reactive oxygen species (ROS), a type of highly reactive hydroxyl radicals, are increased under PM2.5 stimulation 24, 25. Excessive ROS leads to intracellular signaling alteration, DNA damage, cell apoptosis, and the expression of proinflammatory factors, including TNF-α, IL-1β, IL-6, IL-8, and monocyte chemoattractant protein-1 2. In addition, ROS is a critical factor in EMT progression. PM2.5 enhances transforming growth factor β/small mothers against decapentaplegic 2/3 signaling-dependent EMT by increasing ROS levels in cells 26. Although we did not detect ROS or EMT marker levels in this study, the increased levels of prometastatic molecules pro-MMP-2 (Figure 2) and ICAM-1 (Figure 3) indicate that PM2.5 induces tumor metastatic activity. Therefore, targeting ROS-mediated MMPs and ICAM-1 production may be a new antitumor strategy under PM2.5 exposure.

Previous studies have shown that luteolin, an antioxidant and anti-inflammatory agent 12, could upregulate the expression of pro-apoptotic caspase-3 to induce cell death in breast cancer MDA-MB-231 cells 27. Luteolin is also reported to inhibit MMPs secretion and EMT activity in pancreatic cancer cells 28. In addition, luteolin could modulate nuclear factor erythroid 2-related factor 2 (Nrf2) pathway to block colorectal carcinogenesis *in vitro* and *in vivo* 29, 30. Furthermore, by inhibiting Nrf2 activity, luteolin increases the sensitivity of non-small cell lung cancer A549 cells in various anticancer drugs 31. In this study, we found that luteolin treatment considerably reduces the activity of pro-MMP-2 (Figure 2) and ICAM-1 (Figure 3), suggesting that luteolin exerts anti-metastatic ability.

Many natural compounds have been used to treat cancers 10. Jia et al. reveal that quercetin could suppress the progression of breast cancer by inducing AKT/mTOR pathway-mediated autophagy 32. In colorectal cancer and hepatocellular carcinoma, kaempferol blocks ROS production and impedes cell migration 33. Chrysin from honey and quercetin from onions are also suggested to modulate the accumulation of hypoxia-inducible factor-1α which subsequently affect the carcinogenesis and cancer progression 34, 35. In this study, the application of luteolin effectively reduces the expressions of PM2.5-induced pro-metastatic factors, such as pro-MMP-2 and ICAM-1, in human lung cancer H460 cells. Thus, luteolin is worthy of consideration to be used on controlling tumor progression.

**Figure 8:**
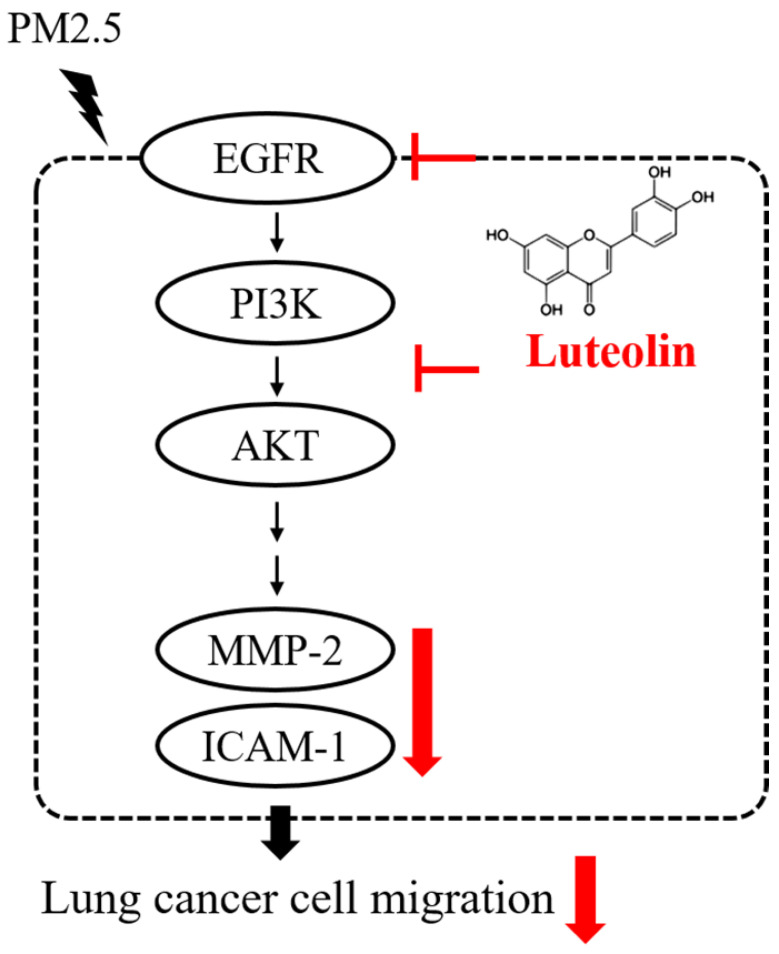
** Conclusion of this study.** Luteolin blocks PM2.5-induced EGFR-PI3K-AKT signaling and subsequently reduces the expression of MMP-2 and ICAM-1. Therefore, the migration ability of human lung cancer H460 cells is inhibited. Collectively, luteolin is considered as a potent therapy of lung cancer progression.

In summary, the productions of pro-MMP-2 and ICAM-1, as well as the capability of cell migration, were reduced by luteolin in PM2.5-stimulated H460 cells. Luteolin also blocks the PM2.5-prompted EGFR-PI3K-AKT pathway (Figure 8). Our investigation demonstrates remarkable inhibitory effects of luteolin on tumor progression, indicating that luteolin is a potential anticancer therapy.

## Supplementary Material

Supplementary table.

## References

[1] Loomis D, Huang W, Chen G (2014). The International Agency for Research on Cancer (IARC) evaluation of the carcinogenicity of outdoor air pollution: focus on China. Chin J Cancer.

[3] Apte JS, Marshall JD, Cohen AJ, Brauer M (2015). Addressing Global Mortality from Ambient PM2.5. Environ Sci Technol.

[4] Cohen AJ, Brauer M, Burnett R, Anderson HR, Frostad J, Estep K (2017). Estimates and 25-year trends of the global burden of disease attributable to ambient air pollution: an analysis of data from the Global Burden of Diseases Study 2015. Lancet.

[5] Wei H, Liang F, Cheng W, Zhou R, Wu X, Feng Y (2017). The mechanisms for lung cancer risk of PM2.5: Induction of epithelial-mesenchymal transition and cancer stem cell properties in human non-small cell lung cancer cells. Environ Toxicol.

[6] Lee H, Hwang-Bo H, Ji SY, Kim MY, Kim SY, Park C (2020). Diesel particulate matter2.5 promotes epithelial-mesenchymal transition of human retinal pigment epithelial cells via generation of reactive oxygen species. Environ Pollut.

[7] Xu Z, Ding W, Deng X (2019). PM2.5, Fine Particulate Matter: A Novel Player in the Epithelial-Mesenchymal Transition?. Front Physiol.

[8] Liu CW, Lee TL, Chen YC, Liang CJ, Wang SH, Lue JH (2018). PM2.5-induced oxidative stress increases intercellular adhesion molecule-1 expression in lung epithelial cells through the IL-6/AKT/STAT3/NF-kappaB-dependent pathway. Part Fibre Toxicol.

[9] Liang S, Zhao T, Hu H, Shi Y, Xu Q, Miller MR (2019). Repeat dose exposure of PM2.5 triggers the disseminated intravascular coagulation (DIC) in SD rats. Sci Total Environ.

[10] Ponte LGS, Pavan ICB, Mancini MCS, da Silva LGS, Morelli AP, Severino MB (2021). The Hallmarks of Flavonoids in Cancer. Molecules.

[11] Lin Y, Shi R, Wang X, Shen HM (2008). Luteolin, a flavonoid with potential for cancer prevention and therapy. Curr Cancer Drug Targets.

[12] Shimada H, Miura K, Imamura Y (2006). Characteristics and inhibition by flavonoids of 20alpha-hydroxysteroid dehydrogenase activity in mouse tissues. Life Sci.

[13] Birt DF, Hendrich S, Wang W (2001). Dietary agents in cancer prevention: flavonoids and isoflavonoids. Pharmacol Ther.

[14] Jia Z, Nallasamy P, Liu D, Shah H, Li JZ, Chitrakar R (2015). Luteolin protects against vascular inflammation in mice and TNF-alpha-induced monocyte adhesion to endothelial cells via suppressing IKappaBalpha/NF-kappaB signaling pathway. J Nutr Biochem.

[15] Yang H, Liu Q, Ahn JH, Kim SB, Kim YC, Sung SH (2012). Luteolin downregulates IL-1beta-induced MMP-9 and -13 expressions in osteoblasts via inhibition of ERK signalling pathway. J Enzyme Inhib Med Chem.

[16] Zhang W, Jiang P, Chen J, Zhu C, Mao Z, Gao C (2017). Application of melatonin-loaded poly(N-isopropylacrylamide) hydrogel particles to reduce the toxicity of airborne pollutes to RAW264.7 cells. J Colloid Interface Sci.

[17] Kuo CY, Wang JY, Chang SH, Chen MC (2009). Study of metal concentrations in the environment near diesel transport routes. Atmospheric Environment.

[18] Feng S, Gao D, Liao F, Zhou F, Wang X (2016). The health effects of ambient PM2.5 and potential mechanisms. Ecotoxicol Environ Saf.

[19] Bethune G, Bethune D, Ridgway N, Xu Z (2010). Epidermal growth factor receptor (EGFR) in lung cancer: an overview and update. J Thorac Dis.

[20] Cheng H, Shcherba M, Pendurti G, Liang Y, Piperdi B, Perez-Soler R (2014). Targeting the PI3K/AKT/mTOR pathway: potential for lung cancer treatment. Lung Cancer Manag.

[21] Kostler WJ, Tomek S, Brodowicz T, Budinsky AC, Flamm M, Hejna M (2001). Soluble ICAM-1 in breast cancer: clinical significance and biological implications. Cancer Immunol Immunother.

[22] Duzagac F, Inan S, Ela Simsek F, Acikgoz E, Guven U, Khan SA (2015). JAK/STAT pathway interacts with intercellular cell adhesion molecule (ICAM) and vascular cell adhesion molecule (VCAM) while prostate cancer stem cells form tumor spheroids. J BUON.

[23] Lewandowska AM, Rudzki M, Rudzki S, Lewandowski T, Laskowska B (2019). Environmental risk factors for cancer - review paper. Ann Agric Environ Med.

[25] Loomis D, Grosse Y, Lauby-Secretan B, El Ghissassi F, Bouvard V, Benbrahim-Tallaa L (2013). The carcinogenicity of outdoor air pollution. Lancet Oncol.

[26] Dysart MM, Galvis BR, Russell AG, Barker TH (2014). Environmental particulate (PM2.5) augments stiffness-induced alveolar epithelial cell mechanoactivation of transforming growth factor beta. PLoS One.

[27] Huang L, Jin K, Lan H (2019). Luteolin inhibits cell cycle progression and induces apoptosis of breast cancer cells through downregulation of human telomerase reverse transcriptase. Oncol Lett.

[28] Huang X, Dai S, Dai J, Xiao Y, Bai Y, Chen B (2015). Luteolin decreases invasiveness, deactivates STAT3 signaling, and reverses interleukin-6 induced epithelial-mesenchymal transition and matrix metalloproteinase secretion of pancreatic cancer cells. Onco Targets Ther.

[29] Zuo Q, Wu R, Xiao X, Yang C, Yang Y, Wang C (2018). The dietary flavone luteolin epigenetically activates the Nrf2 pathway and blocks cell transformation in human colorectal cancer HCT116 cells. J Cell Biochem.

[30] Pandurangan AK, Ananda Sadagopan SK, Dharmalingam P, Ganapasam S (2014). Luteolin, a bioflavonoid inhibits Azoxymethane-induced colorectal cancer through activation of Nrf2 signaling. Toxicol Mech Methods.

[31] Tang X, Wang H, Fan L, Wu X, Xin A, Ren H (2011). Luteolin inhibits Nrf2 leading to negative regulation of the Nrf2/ARE pathway and sensitization of human lung carcinoma A549 cells to therapeutic drugs. Free Radic Biol Med.

[32] Jia L, Huang S, Yin X, Zan Y, Guo Y, Han L (2018). Quercetin suppresses the mobility of breast cancer by suppressing glycolysis through Akt-mTOR pathway mediated autophagy induction. Life Sci.

[33] Choi JB, Kim JH, Lee H, Pak JN, Shim BS, Kim SH (2018). Reactive Oxygen Species and p53 Mediated Activation of p38 and Caspases is Critically Involved in Kaempferol Induced Apoptosis in Colorectal Cancer Cells. J Agric Food Chem.

[34] Fu B, Xue J, Li Z, Shi X, Jiang BH, Fang J (2007). Chrysin inhibits expression of hypoxia-inducible factor-1alpha through reducing hypoxia-inducible factor-1alpha stability and inhibiting its protein synthesis. Mol Cancer Ther.

[35] Samec M, Liskova A, Koklesova L, Mersakova S, Strnadel J, Kajo K (2021). Flavonoids Targeting HIF-1: Implications on Cancer Metabolism. Cancers (Basel).

