# Supplementary material for: Luteolin Reduces Aqueous Extract PM2.5-induced Metastatic Activity in H460 Lung Cancer Cells
Source: Int J Med Sci. 2022 Aug 21;19(10):1502–9. doi: 10.7150/ijms.73947 (PMC9515691; doi:10.7150/ijms.73947)
Supplement: Supplementary file 1 — Supplementary table. [file ijmsv19p1502s1.pdf]

Supplementary Table 1. The analysis of PM2.5 samples in the South District of Taichung City from August 2017 and May 2018.

**Metal**	**Concentration (μg/g PM2.5)**	**Metal**	**Concentration (μg/g PM2.5)**
Ga	0.034	Se	0.110
Ag	< 0.003	In	< 0.003
Cd	0.036	Hg	N.D.
Sn	0.071	Pb	0.769
Ba	0.486	Cu	4.07
B	0.517	Al	8.95
V	0.688	Ca	27.0
Ni	0.521	Zn	7.31
Co	0.020	Fe	25.4
Cr	0.426	Mg	6.10
As	0.154	Mn	3.08

N.D., not detected.
